# Outer membrane vesicle-mediated serum protection in *Aggregatibacter actinomycetemcomitans*

**DOI:** 10.1080/20002297.2020.1747857

**Published:** 2020-04-08

**Authors:** Mark Lindholm, Marjut Metsäniitty, Elisabeth Granström, Jan Oscarsson

**Affiliations:** Oral Microbiology, Department of Odontology, Umeå University, Umeå, Sweden

**Keywords:** *Aggregatibacter actinomycetemcomitans*, serum resistance, outer membrane vesicles, LPS

## Abstract

*Aggregatibacter actinomycetemcomitans* belongs to the HACEK group of fastidious Gram-negative organisms, a recognized cause of infective endocarditis. *A. actinomycetemcomitans* is also implicated in periodontitis, with rapid progress in adolescents. We recently demonstrated that the major outer membrane protein, OmpA1 was critical for serum survival of the *A. actinomycetemcomitans* serotype a model strain, D7SS, and that the paralogue, OmpA2 could operate as a functional homologue to OmpA1 in mediating serum resistance. In the present work, an essentially serum-sensitive *ompA1 ompA2* double mutant *A. actinomycetemcomitans* strain derivative was exploited to elucidate if *A. actinomycetemcomitans* OMVs can contribute to bacterial serum resistance. Indeed, supplementation of OMVs resulted in a dose-dependent increase of the survival of the serum-sensitive strain in incubations in 50% normal human serum (NHS). Whereas neither OmpA1 nor OmpA2 was required for the OMV-mediated serum protection, OMVs and LPS from an *A. actinomycetemcomitans* strain lacking the LPS O-antigen polysaccharide part were significantly impaired in protecting D7SS *ompA1 ompA2*. Our results using a complement system screen assay support a model where *A. actinomycetemcomitans* OMVs can act as a decoy, which can trigger complement activation in an LPS-dependent manner, and consume complement components to protect serum-susceptible bacterial cells.

## Introduction

The HACEK group of fastidious Gram-negative organisms is an identified cause of infective endocarditis, responsible for 1.4 to 3% of cases [1]. The genus *Aggregatibacter* is now the dominant etiology of HACEK endocarditis [2]. Carriage of *Aggregatibacter actinomycetemcomitans* is strongly associated with periodontitis with rapid progress in adolescents and young adults [3–5]. *A. actinomycetemcomitans* is a genetically diverse species, with the serotypes a-g representing different lineages [5]. *A. actinomycetemcomitans* genotypes that produce high levels of leukotoxin, i.e., JP2 and *cagE*, respectively (serotype b) are strongly linked to periodontal attachment loss progression in North and West African adolescents [4,6]. The systemic role of *A. actinomycetemcomitans*, further to its involvement in endocarditis, includes its association with conditions of soft tissue abscesses, and osteomyelitis [7]. In addition, *A. actinomycetemcomitans* has been identified in atheromatous plaque [8] and is a candidate bacterial trigger of anti-citrulline autoimmunity in rheumatoid arthritis [9,10].

Serum resistance represents an important virulence factor of bacteria that enter into the bloodstream and cause infection, allowing the bacterial cells to evade the innate immune defense mechanisms present in serum, including the complement system and antimicrobial peptides. Mechanisms of bacterial resistance against complement-mediated killing include protection via extracellular polysaccharide capsules, insertion of membrane attack complexes into nonlethal bacterial targets, and expression of factors that inhibit or interfere with the complement cascade [11–14]. Bacterial factors that mediate serum resistance, therefore, represent targets for vaccine and drug development [15,16]. Serum resistance appears to be crucial for *A. actinomycetemcomitans* virulence and is a prevalent feature among strains of this species [17–19]. The outer membrane protein, Omp100 (also referred to as ApiA) was previously shown to be required for full serum resistance of some serotype b, and d *A. actinomycetemcomitans* strains, and to physically interact with the alternative complement pathway negative regulator, Factor H *in vitro* [17,20]. We recently demonstrated that the major outer membrane protein, OmpA1 was critical for serum survival of the *A. actinomycetemcomitans* serotype a model strain, D7SS [21]. Interestingly, serum resistant *ompA1* mutants were fortuitously obtained, which expressed increased levels of the paralogue, OmpA2. Thus, OmpA2, which normally seems to be expressed at low levels *in vitro*, could apparently operate as a functional homologue to OmpA1 in *A. actinomycetemcomitans* serum resistance [21]. As several complement activation pathways, i.e., classical, alternative, and mannose-binding lectin (MBL) complement activation were needed to completely eliminate serum-sensitive *ompA1 ompA2* double mutant *A. actinomycetemcomitans* derivatives [21], it is plausible that serum resistance in this species, similar to in *Acinetobacter baumanii* [22], is highly complex, and depend on multiple gene products.

It has been clearly demonstrated that most Gram-negative bacteria release outer membrane vesicles (OMVs) during normal growth [23,24]. *A. actinomycetemcomitans* OMVs have been shown to deliver virulence factors, such as leukotoxin, and cytolethal distending toxin (CDT) to human cells [25,26], and to internalize into the host cells to act as a trigger of innate immunity [27]. Proteomics, and Western blot analysis of *A. actinomycetemcomitans* OMVs have identified several additional vesicle-associated proteins that can contribute to evasion of the immune defense, including the IL1β-binding lipoprotein, BilRI, Omp100, OmpA1, and OmpA2, and a Factor H-binding protein homologue [21,28,29]. A role of OMVs in contributing to bacterial serum resistance has been demonstrated in a number of bacterial species, including *Moraxella catarrhalis, Neisseria gonorrhoeae, Porphyromonas gingivalis*, and *Vibrio cholerae* [30–33]. Whether *A. actinomycetemcomitans* OMVs may also be involved in serum resistance is not known. A functional role of *A. actinomycetemcomitans* OMVs in complement interaction would be consistent with *in vitro* observations that the vesicles, in an OmpA1- and OmpA2-dependent manner, respectively, could bind to C4-binding protein [21], a major inhibitor of classical and MBL complement activation [34]. Lipopolysaccharide (LPS) is one of the most abundant components of OMVs, including those released by *A. actinomycetemcomitans*, and is displayed on the outer surface of the vesicles [23–25]. It was recently shown that LPS is involved in the binding of IL-8 by *A. actinomycetemcomitans* OMVs [35], and it cannot be excluded that the LPS in OMVs also may interact with complement. The serotype-specific polysaccharide (S-PA) determinant of *A. actinomycetemcomitans* resides in the LPS O antigen, which is immunodominant [36–39]. *A. actinomycetemcomitans* strains are occasionally isolated, which lack the ability to produce the serotype-specific antigen [40]. As speculated previously [40], this may represent a mechanism to evade from antibody-based host responses, which could be advantageous in blood circulation. However, a contradiction with this idea is that the absence of S-PA expression in *A. actinomycetemcomitans* appears to be scarce.

The aim of the present work was to investigate if OMVs may play a role in serum resistance in *A. actinomycetemcomitans*.

## Materials and methods

### Ethics considerations

All procedures were conducted in accordance with the guidelines of the local ethics committee at the Medical Faculty of Umeå University, which are in accordance with the Declaration of Helsinki (64th WMA General Assembly, Fortaleza, October 2013). For the assays using normal human serum (NHS), blood was sampled from healthy volunteers after informed consent.

### Bacterial strains and growth conditions

*A. actinomycetemcomitans* strain D7SS is a naturally genetic competent, smooth-colony derivative of D7S (serotype a), which was originally isolated from a patient with rapidly progressing periodontal disease [41]. An *A. actinomycetemcomitans ompA1 ompA2* double mutant strain, i.e. D7SS *ompA1::spe, ompA2::kan* [Spe^r^, Km^r^] [21] was used as a serum-sensitive test strain. SA3138 is a serotype a *A. actinomycetemcomitans* wild-type strain [40]. SA3139, which was isolated from the same patient as SA3138, carries the serotype a-specific antigen gene cluster, however, does not express the LPS O-antigen polysaccharide, rendering this strain non-serotypeable using immunoassay [40]. DNA from strain D7SS *ompA1* [21] was used in the present work to generate mutant derivatives of SA3138 and SA3139, i.e., SA3138 *ompA1::spe* [Spe^r^], and SA3139 *ompA1::spe* [Spe^r^], respectively. *Escherichia coli* C600 is a prototypical K-12 laboratory strain [42]. Strain SE600 is an *ompA* mutant, generated in C600 S, which is a streptomycin-resistant derivative of C600 [43]. The bacterial strains were routinely cultivated in air supplemented with 5% CO_2_, at 37°C, on blood agar plates (5% defibrinated horse blood, 5 mg hemin/l, 10 mg Vitamin K/l, Columbia agar base). Alternatively, for transformation assays, the strains were grown on Trypticase soy broth supplemented with 0.1% yeast extract, 5% heat-inactivated horse serum, and 1.5% agar (sTSB agar). When needed, the growth media was supplemented with 100 μg/ml (final concentration) kanamycin or spectinomycin, respectively.

### Construction of *ompA1* gene replacement mutants

The *ompA1::spe* allele from D7SS *ompA1* was transferred to strains SA3138 and 3139, using natural transformation [41], generating SA3138 *ompA1* and SA3139 *ompA1*, respectively. Confirmation of the allelic replacements was done by PCR. For this, we used *ompA1*-specific oligonucleotide primers as described earlier [21].

### SDS-PAGE and Western blot analysis

The procedures used for SDS-PAGE and Western blot analysis have been described previously [44,45]. For Western blot, we used normal human serum samples from six periodontally healthy donors. Two sera were shown earlier to exhibit high, and one to exhibit low reactivity, respectively, towards recombinant *A. actinomycetemcomitans* leukotoxin [46]. Moreover, one serum was sampled from an individual who was confirmed to be *A. actinomycetemcomitans*-negative according to cultivation from subgingival plaque [47], albeit exhibited reactivity towards *A. actinomycetemcomitans* peptidoglycan-associated lipoprotein (PAL) [28]. All serum samples tested were used at a final dilution of 1:2,000. As secondary antibody, anti-human horseradish peroxidase (HRP)-conjugate was used (Jackson ImmunoResearch, Newmarket, UK) (1:10,000). Immunoreactive bands were visualized using Clarity™ Western ECL Substrate (Bio-Rad), and the ChemiDoc™ MP imaging system (Bio-Rad).

### Isolation of outer membrane vesicles

OMVs were isolated from *A. actinomycetemcomitans* cells harvested from an average of 10 blood agar plates, using ultracentrifugation as described earlier [26,27]. OMV pellets were washed twice with PBS (85,000 × *g*; 2 h, 4°C) using a 70 Ti rotor (Beckman Instruments Inc.), resuspended in ≈200 μl PBS, and then used as the OMV preparation. The yield of OMVs was estimated by determining protein concentrations using a NanoDrop 1000 spectrophotometer (Thermo Fisher Scientific), and preparations were assessed for OMV particle concentration using Nanoparticle Tracking Analysis software (NanoSight Ltd.). OMVs were tested for the absence of bacterial contamination by cultivating small aliquots on blood agar plates in air supplemented with 5% CO_2_, at 37°C for 3 days.

### Isolation of LPS

LPS was isolated from *A. actinomycetemcomitans* cells harvested from 12 blood agar plates, using a combination of procedures [48,49], as described [35]. LPS preparations were resuspended in 100 µl H_2_O and the yield (typically 1.5–2.8 × 10^6^ endotoxin units [EU]) was estimated using the ToxinSensor™ chromogenic Limulus Amebocyte Lysate (LAL) endotoxin kit (BioNordika, Sweden).

### Bacterial serum sensitivity assay

To determine the sensitivity of *A. actinomycetemcomitans* cells to normal human serum, NHS was taken from healthy volunteers. We essentially followed procedures described previously [21], using *A. actinomycetemcomitans* strains grown on agar. In brief, prior to being used in the assays, bacteria were harvested and suspensions were adjusted to 1.0 × 10^9^ cells/ml in PBS buffer. Reaction mixtures contained 50% NHS, i.e. 105 μl NHS, 95 μl PBS, and 10 μl bacterial suspension, and were incubated at 37°C for 1-2 h. For the serum sensitivity assay with OMVs, the PBS was supplemented with OMVs isolated from *A. actinomycetemcomitans* strains. The PBS was supplemented with vesicles equivalent to 20, 100, and 200 μg protein, respectively, yielding a final concentration of 95.2, 476.2, and 952.4 μg/ml in the reaction mixtures. Alternatively, 50 ng (500 EU) LPS was added to the reaction mixtures. Bacterial serum survival was determined by viable count, and the increase in serum survival upon OMV or LPS supplementation was determined relative to incubations in vesicle-free controls (50% NHS in PBS).

### Assessment of complement consumption by OMVs

To determine the consumption of complement by *A. actinomycetemcomitans* OMVs, vesicles (20 and 100 μg, respectively) in PBS were incubated in 50% NHS at 37°C for 1 h in a reaction volume of 200 μl, yielding final concentrations of 95.2 and 476.2 μg protein/ml. Alternatively, 50 ng (500 EU) LPS was used instead of OMVs. The remaining complement activity in the NHS samples was subsequently quantified using the Wieslab® Complement System Screen kit, COMPL300 (Svar Life Science AB, Malmö, Sweden), according to the instructions of the manufacturer. NHS incubated with PBS alone served as a negative control for complement consumption.

### Statistical analysis and image processing

The statistical significance of the data was calculated using two-tailed Student’s t-test. The level of statistical significance was set to *P* < 0.05, based on at least three independent experiments unless otherwise stated. Images for figures were assembled using Adobe Photoshop CS6, or Microsoft PowerPoint.

## Results

### *OMVs mediate LPS-dependent serum protection of* A. actinomycetemcomitans *cells*

**Figure 1.**
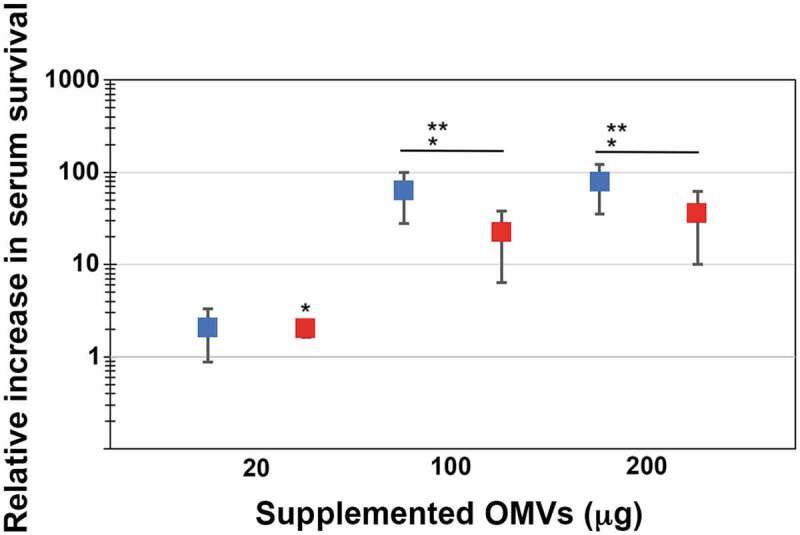
Enhanced serum survival of *A. actinomycetemcomitans* strain D7SS *ompA1 ompA2* upon supplementation of OMVs. *A. actinomycetemcomitans* cells were incubated in 50% normal human serum (NHS) at 37°C for 1 h. The assay was performed in the absence or presence of 20, 100, and 200 μg of OMVs, respectively, as indicated. Bacterial serum survival was determined by viable count, and shown is the increase (fold change) in survival relative to incubations in vesicle-free controls (50% NHS in PBS). Supplemented OMVs were obtained from the wild-type strain D7SS (blue squares), and D7SS *ompA1 ompA2* (red squares), respectively. Shown are means ± SEM from four independent experiments. *P < 0.05 vs control. **P < 0.05 vs supplementation of 20 μg of OMVs. *P* > 0.05, D7SS OMVs vs D7SS *ompA1 ompA2* OMVs

**Figure 2.**
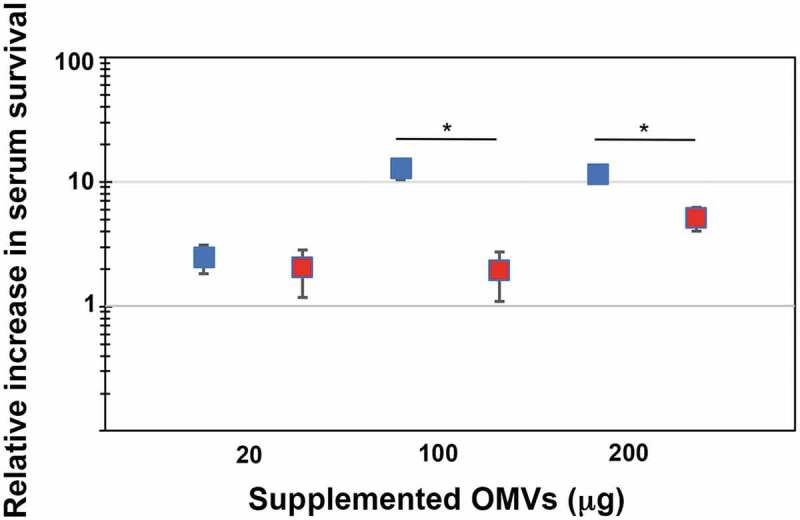
LPS-dependent serum protection of *A. actinomycetemcomitans* strain D7SS *ompA1 ompA2* upon supplementation of OMVs. *A. actinomycetemcomitans* cells were incubated in 50% normal human serum (NHS) at 37°C for 1 h. The assay was performed in the absence or presence of 20, 100, and 200 μg of OMVs, respectively, as indicated. Bacterial serum survival was determined by viable count, and shown is the increase (fold change) in survival relative to incubations in vesicle-free controls (50% NHS in PBS). Supplemented OMVs were obtained from strain SA3138 (serotype a, wildtype; blue squares), and SA3139 (as SA3138 but LPS lacks the O-antigen polysaccharide part; red squares). Shown are means ± SEM from four independent experiments. *P < 0.05 SA3138 OMVs vs SA3139 OMVs

In order to test if outer membrane vesicles may be involved in serum resistance of *A. actinomycetemcomitans*, OMVs were isolated from the wild-type strain, D7SS, and from its *ompA1 ompA2* double mutant derivative, as described in Materials and methods. Total protein concentrations of obtained vesicle preparations were in average ≈6-7 mg/ml, and which contained approximately 1 × 10^9^ OMV particles/ml according to NanoSight analysis. *A. actinomycetemcomitans* strain D7SS *ompA1 ompA2* was utilized in the serum protection assays as it is essentially serum-sensitive [21]. According to our findings (Figure 1), the survival rate of this strain was clearly increased (up to approximately 100-fold) in a dose-dependent manner upon supplementation of D7SS OMVs equivalent to 20 and 100 μg protein. In contrast, there was no further increase in serum survival when D7SS vesicles corresponding to 200 μg protein was supplemented, suggesting that the serum protection was saturated. To investigate whether the protective effect by *A. actinomycetemcomitans* OMVs was OmpA-dependent, the same assay was performed, but instead supplementing OMVs obtained from the *ompA1 ompA2* double mutant strain (Figure 1). According to our results, the *ompA* double mutant OMVs had a slightly, however not statistically significantly reduced, protective effect on the serum survival of D7SS *ompA1 ompA2*. We, therefore, concluded that *A. actinomycetemcomitans* OMVs could mediate serum protection, essentially independent of OmpA. To investigate the potential role of LPS in OMV-mediated serum protection, vesicles were isolated from the *A. actinomycetemcomitans* wild-type strain, SA3138, and from strain SA3139, which has an LPS lacking the O-antigen polysaccharide part. According to our results, OMVs obtained from the LPS O-antigen-deficient strain, SA3139, were significantly attenuated in protecting the D7SS *ompA1 ompA2* double mutant in serum sensitivity assays (Figure 2). We, therefore, concluded that serum protection by *A. actinomycetemcomitans* OMVs was LPS-dependent. A similar result was obtained in serum protection assays using purified LPS instead of OMVs. Upon supplementation of 50 ng (500 EU) LPS, there was a higher (3.4-fold ± 0.375 [SEM]) survival of D7SS *ompA1 ompA2* in presence of SA3138 LPS, compared to when LPS from SA3139 was used instead. On the other hand, strains SA3138 and SA3139 were both found to be serum resistant (survival rate ≥100%), whereas, upon inactivation of the *ompA1* gene in these strains, their serum resistance was essentially lost (survival rate ≤1%), consistent with OmpA1 being a major factor contributing to the intrinsic serum resistance of *A. actinomycetemcomitans* strains [21].

### Complement consumption by *A. actinomycetemcomitans* OMVs is LPS-dependent

As supplementation of *A. actinomycetemcomitans* OMVs protected strain D7SS *ompA1 ompA2* in serum sensitivity assays, we investigated whether the vesicles could activate, and consume serum complement components. To this end, 50% NHS was preincubated with OMVs equivalent to 20 and 100 μg protein, respectively, and then assessed with the COMPL300 assay (Materials and methods) to determine the remaining activity of the classical, alternative, and MBL pathways of complement activation, respectively. According to our results, upon addition of the 20 μg samples, OMVs from D7SS, the D7SS *ompA1 ompA2* double mutant, and SA3138 all efficiently activated complement, and consumed components of the tested activation pathways (Table 1). Using 100 μg OMVs there was <1% residual activity in the three pathways, consistent with complement component titration by the vesicles. In contrast, most of the complement activity in the three pathways were remaining in the NHS preincubated with SA3139 OMVs, lacking the LPS O-antigen polysaccharide part (Table 1). We, therefore, concluded that LPS under the present conditions was a major OMV antigen responsible for complement consumption through the tested activation pathways. A similar result was obtained using LPS, purified from SA3138 and SA3139, i.e. LPS from the latter strain consumed less complement activity (Table 2).

**Table 1.** Remaining complement activity (%) in the indicated activation pathways after consumption of complement in NHS by *A. actinomycetemcomitans* OMVs (20 μg; rendering a final OMV protein concentration of 95.2 μg/ml) for 1 h at 37°C. NHS incubated with PBS served as a negative control. Complement consumption was thereafter determined using the COMPL300 kit, as described in Materials and methods. Values are given as mean percentage (range) from two independent experiments

OMV sample^a^	Classical (%)	MBL (%)	Alternative (%)
D7SS	24.6 (24.5–24.7)	42.5 (41.8–43.2)	2.3 (2.1–2.4)
D7SS Δ*ompA1 ompA2*	26.1 (25.8–26.4)	28.6 (28.0–29.1)	3.7 (3.3–4.2)
SA3138	18.1 (17.7–18.5)	29.4 (28.3–30.5)	2.4 (2.4–2.5)
SA3139	112.1 (110.8–113.4)	138.7 (137.0–140.3)	84.3 (80.6–87.9)
PBS (control)	106.0 (101.9–110.1)	116.3 (114.0–118.6)	96.7 (83.0–110.3)

^a^OMVs isolated from the indicated *A. actinomycetemcomitans* strain were preincubated with 50% NHS.

**Table 2.** Remaining complement activity (%) in the indicated activation pathways after consumption of complement in NHS by *A. actinomycetemcomitans* LPS (50 ng; 500 EU) for 1 h at 37°C. Complement consumption was thereafter determined using the COMPL300 kit, as described in Materials and methods. Values are given as mean percentage (range) from two independent experiments. *P* < 0.05 SA3138 OMVs vs SA3139 OMVs for all three tested pathways

LPS sample^a^	Classical (%)	MBL (%)	Alternative (%)
SA3138	46.8 (50.6–42.9)	15.8 (22.2–9.3)	10.8 (12.4–9.2)
SA3139	67.8 (70.4–65.1)	78.8 (79.3–78.3)	50.8 (58.8–42.8)

^a^LPS isolated from the indicated *A. actinomycetemcomitans* strain were preincubated with 50% NHS.

### Normal human serum of periodontally healthy subjects contains antibodies recognizing *A. actinomycetemcomitans* OMV antigens

**Figure 3.**
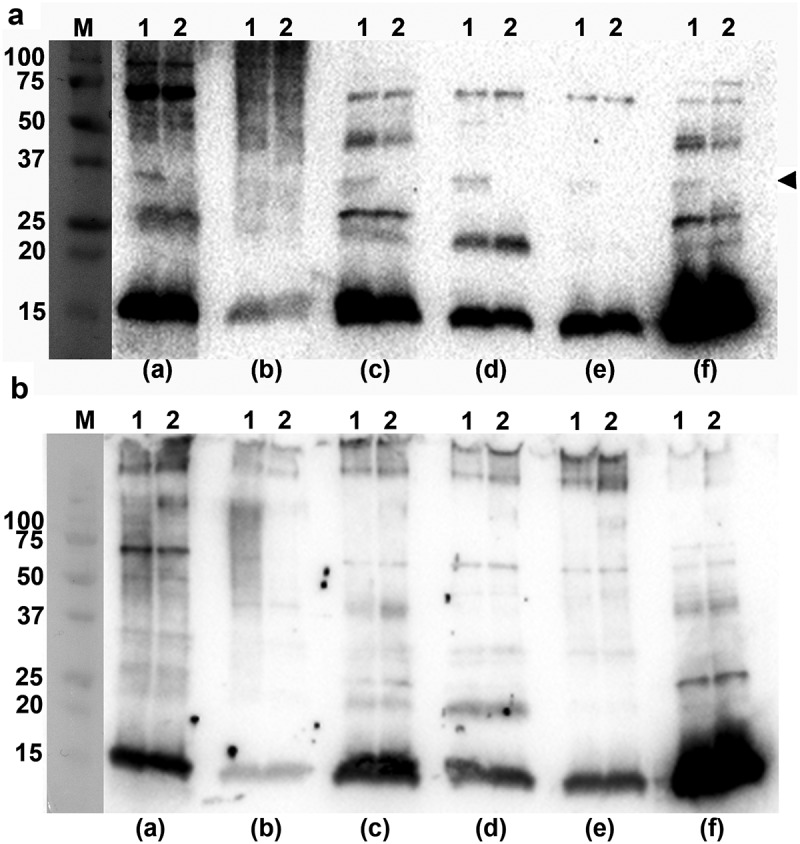
Immunoreactivity of normal human sera to *A. actinomycetemcomitans* and *E. coli* OMVs. Western blot analysis of reactivity of NHS from periodontally healthy individuals with (a) OMVs obtained from the *A. actinomycetemcomitans* strains D7SS (wild-type; lane 1), and D7SS *ompA1 ompA2* (lane 2), and (b) OMVs obtained from the *A. actinomycetemcomitans* strains SA3138 (wild-type; lane 1), and SA3139 (LPS lacks the O-antigen polysaccharide part; lane 2). The NHS samples a-f were used for immunodetection as indicated, and include sera known to exhibit high (a, and f) and low (b) reactivity, respectively, towards recombinant *A. actinomycetemcomitans* leukotoxin, and one from a confirmed *A. actinomycetemcomitans*-negative individual (e). Samples equal to 10 μg protein were applied on the gels. The reactive band corresponding to *A. actinomycetemcomitans* OmpA1 is indicated with an arrowhead. LPS is detected as a diffuse, high-molecular smear pattern for serum sample (b), when assessing SA3138 OMVs. The sizes (kDa) of the proteins in the pre-stained molecular weight marker (M) are indicated along the left sides

Albeit classical complement consumption by *A. actinomycetemcomitans* OMVs to a large extent was OmpA-independent (Table 1), we wanted to clarify whether normal human serum may contain antibodies recognizing *A. actinomycetemcomitans* OMV antigens, which could play a role in the classical complement activation by the vesicles. To this end, Western blot was used to assess a selection (n = 6) of NHS from periodontally healthy subjects for their ability to recognize antigens of OMVs obtained from *A. actinomycetemcomitans* strains. Comparing the reactivity of the sera towards OMVs isolated from D7SS, and D7SS *ompA1 ompA2*, respectively (Figure 3(a)), revealed that all sera, including one sampled from a confirmed, *A. actinomycetemcomitans*-negative individual, recognized species-specific antigens, although at varying degrees. We concluded that *A. actinomycetemcomitans* OmpA1 was recognized by at least five of the six tested sera, as a reactive band specific for this protein was not detected when assessing OMVs from the D7SS *ompA1 ompA2* double mutant. It cannot be excluded that such antibodies have been developed against OMPs and/or surface proteins of commensals, as *E. coli* OMV antigens, including OmpA, were recognized by at least four of the six tested sera (Supplementary Figure 1). A similar pattern of antigen recognition was observed comparing OMVs obtained from the serotype a wild-type strain, SA3138, and the LPS O-antigen-deficient strain SA3139 (Figure 3(b)). In Western blots, the serotype-specific antigen of *A. actinomycetemcomitans* LPS is typically detected as a diffuse smear pattern [40,50], also when assessing OMV samples [28]. This type of banding pattern was detected for SA3138 OMVs when testing serum sample (b), but not for OMVs from the O-antigen deficient SA3139. Hence, serum (b) was most likely sampled from an individual carrying a serotype a *A. actinomycetemcomitans*. Collectively, these observations are in accordance with the notion that there are antibodies present in NHS of periodontally healthy subjects, which can recognize *A. actinomycetemcomitans* OMV antigens, to trigger classical complement activation. However, our results are also consistent with an apparent antibody-independent complement activation by LPS in *A. actinomycetemcomitans* OMVs.

## Discussion

In this work, we have used a highly serum-sensitive *ompA1 ompA2* double mutant *A. actinomycetemcomitans* strain to demonstrate that OMVs play a role in serum protection in this organism. Such a function of *A. actinomycetemcomitans* OMVs is in agreement with findings with OMVs of a number of other bacterial species, i.e. *M. catarrhalis, N. gonorrhoeae, P. gingivalis*, and *V. cholerae* [30–33].

Based on previous studies with *A. actinomycetemcomitans* [17,21,51], we routinely determined the serum survival in 50% NHS. *A. actinomycetemcomitans* strains of all serotypes are ubiquitously serum resistant [17,19], and due to the apparent unavailability of a natural serum-susceptible strain, a strain D7SS *ompA1 ompA2* double mutant was selected for the present experimental studies. This derivative exhibits an essentially abolished serum resistance and is susceptible to the classical, alternative, and MBL pathways of complement activation, whereas its survival rate is similar to the wild-type, D7SS, in heat-inactivated NHS [21]. The serum sensitivity of the double mutant is likely accompanied by at least a partially impaired membrane stability, as electron microscopy revealed larger membrane-vesicle like structures occasionally projecting from the cell surface, and the strain has also a somewhat reduced growth rate in liquid cultures [21].

From the present work, we could conclude that *A. actinomycetemcomitans* OMVs contributed to serum protection, and strongly activated, and consumed the components of all three tested complement activation pathways. Activation of the classical complement pathway by the vesicles is consistent with our current observations that naturally occurring antibodies in NHS can recognize *A. actinomycetemcomitans* OMV antigens, including OmpA1. However, classical complement activation has also been demonstrated to be induced by bacterial OMPs and/or surface proteins in an antibody-independent manner [52–54]. We observed that OmpA1 and OmpA2 were not critical for serum protection and complement consumption by the OMVs. This was somewhat unexpected as these OMPs were necessary for the serum resistance of strain D7SS and bound to recombinant C4-binding protein in *in vitro* incubations with *A. actinomycetemcomitans* OMVs [21]. Thus, *A. actinomycetemcomitans* OMVs appeared to mediate serum protection via a different mechanism than, e.g., *V. cholerae* OMVs, which were found to divert naturally occurring antibodies against Gram-negative organisms away from the bacterial cells via OmpU on the released vesicles [30]. Also, OMVs of *M. catarrhalis* and *N. gonorrhoeae* seemingly depended on OMPs when contributing to serum resistance [31,32]. It can be speculated that the requirement of OMPs for OMV-mediated serum protection might be stronger in some bacterial species, such as *Neisseria meningitidis*, where LPS has a weak complement-activating capability [55].

In the present work, we compared the serum protection efficiency of OMVs, and LPS, derived from the *A. actinomycetemcomitans* serotype a strains SA3138 and SA3139, which were originally isolated from the same individual, but with the latter strain not expressing the LPS O-antigen polysaccharide part [40]. Our observation that SA3139 OMVs, and LPS, only weakly activated complement through any of the tested pathways is consistent with a major role of the LPS O-antigen polysaccharide in the complement activation and consumption by *A. actinomycetemcomitans* OMVs. Concomitantly, and most likely as a consequence of their modest level of complement consumption, OMVs and LPS derived from SA3139, as compared to SA3138, had a clearly lower ability to protect the highly serum-sensitive strain D7SS *ompA1 ompA2* double mutant in 50% NHS. Hence, our present results support a model in which *A. actinomycetemcomitans* OMVs contribute to serum protection as a complement target, which in an LPS-dependent manner can consume and titrate complement components to prevent them from interacting with the bacterial cells. LPS-dependent serum protection by OMVs has been demonstrated previously, for vesicles released by the periodontal pathogen *P. gingivalis* [33]. Such function of *A. actinomycetemcomitans* OMVs is plausible, considering the efficient consumption of complement components by LPS of this species [56]. Moreover, LPS derived from a number of Gram-negative bacteria can block the serum bactericidal activity [33,57], and interact with the classical complement factor C1q, in an antibody-independent fashion, to activate the classical pathway [58,59]. LPS is also a well-recognized alternative complement activator [60], and furthermore, it can trigger the MBL pathway [61].

The potential role *in vivo* of OMV-mediated serum protection is not known. Evidently, bacteria release OMVs during infection *in vivo*, and vesicles have been detected in, e.g., fluids from infected hosts, demonstrating their ability to disseminate distant from the site of infection [32,62,63]. Notably, using a mouse model, it was recently demonstrated that *A. actinomycetemcomitans* OMVs can pass the blood-brain barrier [64]. Bacterial production of OMVs, and their composition is influenced by various environmental factors and sources of cellular stress, which bacterial pathogens experience inside the host [23,24,65]. Evidence from electron microscopy supports that OMV production can be increased upon exposure to host components such as serum, and tissues [23,66,67]. Increased vesicle production appears to improve bacterial survival under stress [68], and higher levels of released vesicles may be associated with virulence, e.g., in leukotoxic as compared to non-leukotoxic *A. actinomycetemcomitans* strains [65,69]. It remains, however, to be determined if vesicle production during bacterial infections on occasions can reach levels *in vivo* to make a significant contribution in serum protection of complement-susceptible bacteria. Complement consumption by OMVs may represent a strategy by pathogens in which they collaborate to conquer innate immunity. The idea of interbacterial serum protection by OMVs is supported by observations that *M. catarrhalis* vesicles can protect *Haemophilus influenzae* from complement-mediated killing *in vitro* [32]. Furthermore, interestingly, *P. gingivalis* vesicles protected two serum-sensitive oral species *in vitro*, i.e., *Bacteroides loeschii* and *Capnocytophaga ochracea* [33], suggesting the possibility that OMV-mediated serum protection could favor the pathogenic progress of periodontitis.

In summary, the present work has revealed a role of OMVs in contributing to *A. actinomycetemcomitans* serum protection, by acting as a decoy, consuming complement in an LPS-dependent fashion. Detailed comprehension of OMV-host interactions, which mediate serum protection may advance the development of tailor-made OMV-based vaccines, agents that reduce bacterial serum resistance, and/or that can target bacteria that have hitherto evaded vaccination achievements.
